# Artificial Intelligence in Ophthalmology: Practical Applications, Subspecialty Evidence and Real-World Deployment

**DOI:** 10.7759/cureus.96121

**Published:** 2025-11-05

**Authors:** Arham Yahya Rizwan Khan, Muhammad Bilal Malik

**Affiliations:** 1 Medicine and Surgery, Shifa International Hospitals Limited, Islamabad, PAK; 2 Ophthalmology, University of Calgary, Calgary, CAN

**Keywords:** artificial intelligence (ai), artificial intelligence in healthcare, deep learning artificial intelligence, machine learning, ophthalmology

## Abstract

Artificial Intelligence (AI) has undoubtedly emerged as a transformative technology in the field of medicine. In ophthalmology, it has been a catalyst for innovation in the methods used for the diagnosis, management, and treatment of different eye diseases. This article offers a detailed review of the literature on the application and utilization of AI technology in the field of ophthalmology.

A detailed search of available literature on the use of AI in the field of ophthalmology was performed through the PubMed database and Google Scholar. Published literature on the role of AI in screening, diagnosis, and management of common ocular conditions such as diabetic retinopathy (DR), cataract, glaucoma, and age-related macular degeneration (AMD) was reviewed. Special emphasis was laid on the effectiveness and limitations of the recently developed AI systems for the detection and management of ocular conditions. We screened (n=4449) records and included (n=102) studies spanning retina, glaucoma, cornea, pediatric ophthalmology, neuro-ophthalmology, ocular oncology, surgery, emergencies, and tele-ophthalmology.

Deep learning (DL) and machine learning (ML) algorithms have demonstrated significant performance in the analysis of ophthalmic data, including optical coherence tomography scans and retinal images, for accurately diagnosing and classifying diseases, predicting disease progression, and personalizing different treatment plans. In addition to the common ocular conditions, the use of AI has now spread to other domains of ophthalmology, such as pediatric ophthalmology, oculoplastics and reconstructive surgery, and triage and management of emergency ocular conditions. Various AI systems have shown accuracy similar to that of clinical experts, with the additional benefit of being less subjective and time-consuming. Despite significant progress, different challenges related to regulatory approval, standardization, data quality, and ethical considerations hamper the wide-scale implementation of AI in ophthalmology.

Literature is evident on the transformative role of AI in screening, diagnosis, and management of various ocular conditions. However, currently, there are various challenges and limitations to the implementation of AI. Future research should focus on addressing these challenges while optimizing the utilization of AI algorithms for enhancing patient care in ophthalmology.

## Introduction and background

Artificial intelligence refers to computational methods that learn patterns from data to support clinical decisions. In ophthalmology, deep learning has progressed from retrospective image classification to prospective deployment in screening programs and clinics, especially for diabetic retinopathy, glaucoma, age-related macular degeneration, and surgical video analysis [1-4]. This review emphasizes clinically anchored evidence, device and workflow dependencies, and real-world performance rather than generic claims about artificial intelligence in medicine. Various AI algorithms have been seen to enhance the diagnostic and screening accuracy of investigations for various pathologies and are also useful in determining the ideal management plans [5]. There is ongoing research in the fields of medicine and surgery to assess the applicability of AI tools in the diagnosis and management of disease, with an overall emphasis on improved patient care [6].

In recent years, there has been a steady increase in the burden of ocular diseases, including those involving visual impairment and blindness. This ever-increasing burden highlights the need for innovative solutions to enhance the efficiency and accuracy of the current standards of ophthalmic care [7]. Conventionally, ophthalmic diagnosis has been based on the interpretation of clinical symptoms and imaging by medical professionals [8]. Realistically speaking, this approach is prone to human error and subjective variability, thus leading to higher misdiagnosis rates and inappropriate referrals, ultimately leading to patient harm [9]. The invention of technologies such as machine learning (ML) has given rise to ways to automate the process of diagnosis and treatment planning based on the analysis of a large amount of data. Through this automation, the process of diagnosis and subsequent management can be made more efficient [10]. ML is the process by which AI learns from data and writes its own programming to perform a task. Deep learning (DL) is a type of machine learning that uses multiple algorithms that replicate neural connectivity [10]. DL is based on artificial neuronal networks that utilize multiple layers to exhibit higher-level functions from raw data input. In the healthcare industry, DL is used in the analysis of medical imaging data to detect different clinical conditions [11]. When used in the field of ophthalmology, it can help in the screening and identification of major ophthalmic public health diseases such as diabetic retinopathy, glaucoma, retinopathy of prematurity, and various other ocular conditions [12].

Although the concept of AI was initially introduced in 1950, it was not until the mid-2000s that AI began to influence the healthcare industry [13,14]. As with other fields of medicine, AI was readily incorporated into the clinical practice of ophthalmology. The previous decade marked the rapid development of AI applications in ophthalmology, combined with advancements in imaging techniques, data analytics, and computational power [15,16]. AI models can be trained through DL to utilize large data sets of ophthalmic images and clinical details to identify complex patterns, make accurate estimations about disease diagnosis, and help in making the right management choices. Although significant progress has been made, there are still several challenges related to the adoption and implementation of AI in ophthalmology. This narrative review includes [n=102] studies identified through a structured, reproducible search with predefined eligibility criteria and qualitative bias appraisal [17-21]. These challenges include ensuring data quality, standardization, regulatory approval for AI incorporation into clinical practice, transparency, interpretability, and data privacy concerns [22]. There is a need to develop interdisciplinary collaboration between ophthalmologists, computer scientists, and regulatory bodies to address the required challenges and ensure the responsible integration of advanced technology in medical practices [23].

We present methods aligned to Preferred Reporting Items for Systematic Reviews and Meta-Analyses (PRISMA) 2020, results organized by subspecialty with comparative tables, and a discussion that foregrounds limitations, bias risks, and translational gaps.

Methods

We conducted a structured narrative scan of PubMed and Google Scholar for peer-reviewed studies of artificial intelligence in Ophthalmology at all instances published from 1 January 2018 to 15 August 2025. Search strings combined ophthalmic subspecialties and modalities with terms such as “deep learning”, “machine learning”, “autonomous”, “surgical video”, “teleophthalmology”, and “electrophysiology”. Inclusion focused on clinical or clinically adjacent evaluations with quantitative performance metrics, external or prospective validation where available, or health-service outcomes. We cross-checked seminal reporting standards to guide the synthesis and highlight gaps relevant to clinical translation, namely PRISMA 2020 for transparent reporting of evidence syntheses, Consolidated Standards of Reporting Trials-Artificial Intelligence (CONSORT-AI) and Standard Protocol Items: Recommendations for Interventional Trials-Artificial Intelligence (SPIRIT-AI) for trials of AI interventions, and Developmental and Exploratory Clinical Investigations of Decision Support Systems Driven by Artificial Intelligence (DECIDE-AI) for early-stage clinical evaluations [17-20]. We did not perform a meta-analysis. Reporting follows PRISMA 2020 for narrative syntheses, with eligibility criteria, screening counts, risk-of-bias domains, and a PRISMA flow diagram provided.

**Figure 1:**
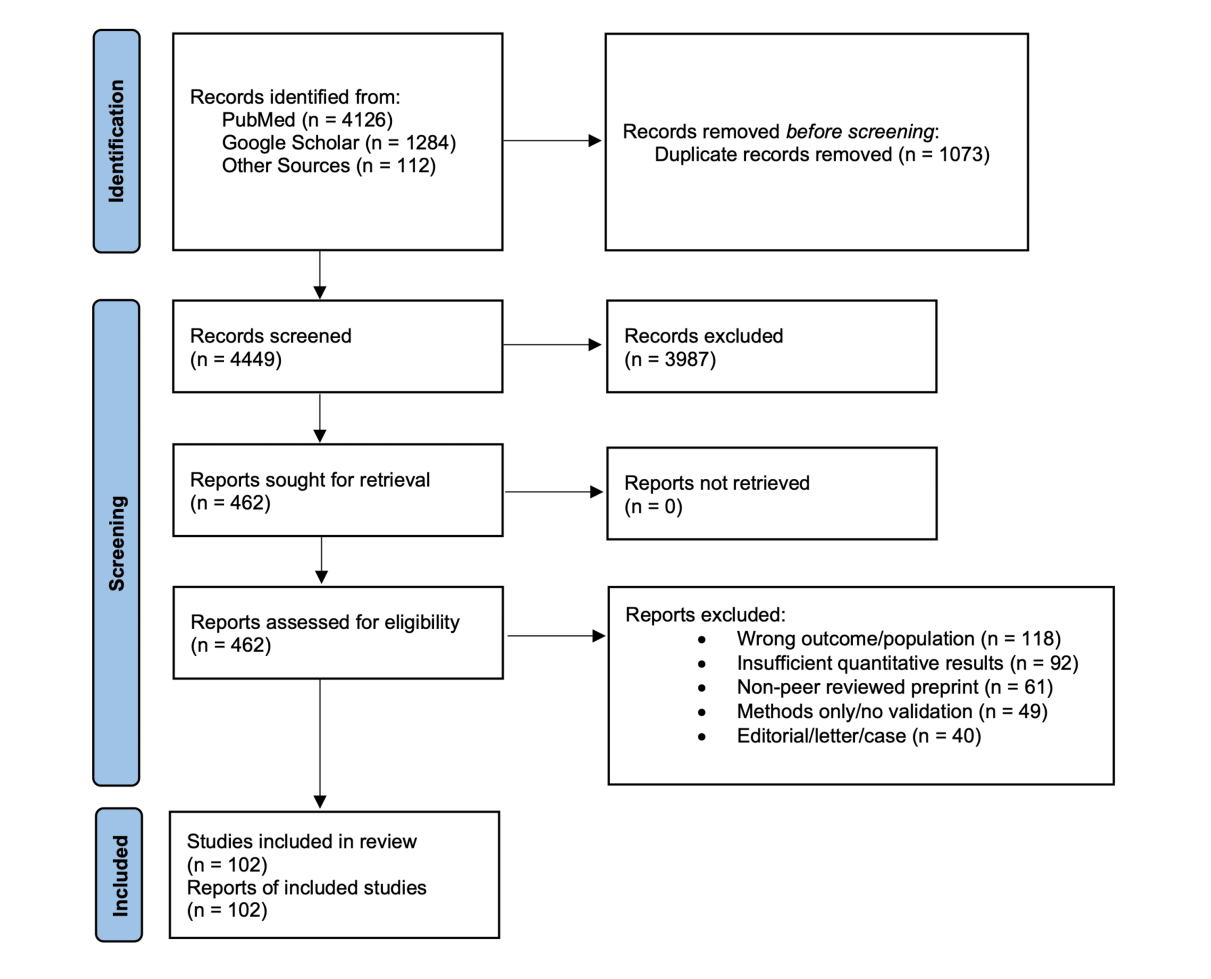
PRISMA 2020 flow diagram with databases Screening, eligibility and final study counts for 1 January 2018 to 15 August 2025.

*Search Strategy and Study Selection* We searched PubMed and Google Scholar from 1 January 2018 to 15 August 2025 using controlled vocabulary and keywords for ophthalmic subspecialties combined with artificial intelligence terms. Two reviewers independently screened titles and abstracts, then full texts, with third-party adjudication for disagreements. We identified 5,522 records in total (PubMed 4,126, Google Scholar 1,284, other sources 112), removed 1,073 duplicates, and screened 4,449 titles and abstracts. We assessed 462 full texts for eligibility and included 102 studies in the narrative synthesis. A standard PRISMA 2020 flow diagram with reasons for exclusion is provided as Figure 1 [17,18].

Eligibility Criteria

As shown in Table 1, we included peer-reviewed primary studies and systematic reviews that evaluated artificial intelligence systems in an ophthalmic clinical or clinically adjacent setting, reported quantitative performance or clinical outcomes, and used human data. We excluded editorials, opinions, case reports, preprints without peer review, studies without sufficient quantitative results, and studies limited to animal or phantom data. For diagnostic accuracy, we prioritized studies reporting area under the receiver operating characteristic curve, sensitivity, specificity, or calibration. Prospective and external validations were noted when present.

**Table 1:** Eligibility criteria and rationale

Domain	Include	Exclude	Rationale
Study type	Clinical primary studies, prospective or retrospective; systematic reviews	Editorials, letters, case reports, non-peer-reviewed preprints	Ensure evaluable evidence
Data	Human participants or human-derived images/signals	Animal, phantom-only studies	Clinical relevance
Outcomes	Diagnostic/prognostic performance, calibration, clinical or service outcomes	Descriptive studies without quantitative metrics	Comparability
Validation	Internal split or cross-validation; note external or prospective validation when present	Pipeline descriptions with no validation	Guard against overfitting
Access/ethics	Peer-reviewed English publications	Non-English if not translatable at screening	Feasible appraisal

Data Extraction and Overlap Management

Two reviewers independently extracted study characteristics into a pre-specified template capturing clinical task, setting, device, and imaging protocol, cohort size, reference standard, validation design, headline metrics with 95% CIs where reported, and calibration or decision-curve metrics. When multiple reports used overlapping datasets, we prioritized the most comprehensive peer-reviewed analysis and did not double-count participants across Results. Disagreements were resolved by consensus.

Risk of Bias and Reporting Quality

We qualitatively appraised included studies with domains adapted from PRISMA 2020 for evidence syntheses, and artificial intelligence-specific guidance for clinical trials and early deployments (CONSORT-AI, SPIRIT-AI, DECIDE-AI) [18-20]. For diagnostic accuracy studies, we referenced the Quality Assessment of Diagnostic Accuracy Studies-Artificial Intelligence (QUADAS-AI) framework to flag artificial intelligence-specific risks, including spectrum bias, reference-standard quality, data leakage, and domain shift [21].

Study-Level Risk of Bias

For each included study, we assessed five domains adapted from QUADAS-AI and AI extensions of CONSORT and SPIRIT: (1) participant spectrum and case mix; (2) reference standard quality; (3) data leakage and overfitting safeguards; (4) device and acquisition transparency; and (5) validation design and calibration reporting. Each domain was graded as low, some concerns, or high risk, with rationale recorded in a structured extraction sheet (Table 2) [21,24-26].

**Table 2:** Study-level risk-of-bias domains adapted from QUADAS-AI and AI-specific CONSORT/SPIRIT extensions with example judgments DR: diabetic retinopathy, QC: quality control.

Domain	Definition	Example judgement for this review	What readers should look for
Spectrum and case mix	Participants reflect the target population	e.g., Many DR datasets from screening rather than primary care; limited pediatric data	Setting and prevalence
Reference standard	Quality of labels and adjudication	e.g., Dual grading with adjudication vs single grader	Adjudication and intergrader agreement
Leakage safeguards	Separation of train/validation/test, no patient-level leakage	e.g., Patient-level rather than image-level splits	Unit of analysis
Device/acquisition	Camera or scanner, field of view, mydriasis, QC	e.g., Several studies lacked device detail	Hardware and protocol
Validation and calibration	External test set, prospective design, calibration/decision curves	e.g., Few studies reported calibration	Prospective external validation and calibration

Synthesis Plan

Given heterogeneity in clinical tasks, datasets, and performance metrics, we conducted a structured narrative synthesis by subspecialty. We reported headline metrics as provided by the authors. Where multiple thresholds were reported, we favored the clinically pre-specified operating point. No meta-analysis was undertaken in accordance with PRISMA 2020 guidance for non-poolable evidence [17].

Device Reporting

For image-based studies, we extracted the acquisition device and protocol when reported, including camera model, field of view, mydriasis policy, image grading protocol, and any embedded quality control. Reporting these parameters is necessary because diagnostic accuracy varies with image quality, field coverage, and hardware domain shift [1-3,24,25].

Summary Metrics and Analysis

For diagnostic accuracy reports, we extracted area under the curve (AUC) with 95% confidence intervals (CIs) when available, sensitivity and specificity at the study’s pre-specified operating point, and any calibration or decision-curve metrics. For prognostic or workflow studies, we recorded hazard ratios, odds ratios, or absolute differences with 95% CIs where reported. Given outcome and design heterogeneity, we present a structured narrative synthesis by subspecialty without quantitative pooling, consistent with PRISMA 2020 guidance for non-meta-analytic reviews [17].

Heterogeneity and Pooling Decision

Clinical tasks, reference standards, and outcome definitions varied across studies. Device heterogeneity and threshold effects precluded meaningful meta-analysis for most subspecialties. For diagnostic accuracy, a hierarchical summary receiver-operating-characteristic or bivariate random-effects model would be appropriate only for homogeneous endpoints with consistent thresholds and devices [27,28]. Given the above, we present a structured narrative comparative synthesis with explicit device and workflow context, in line with PRISMA 2020 guidance for non-poolable evidence [1].

## Review

Use of AI in the detection of diabetic retinopathy

Diabetic retinopathy (DR) is a commonly encountered ophthalmological condition that occurs in diabetic patients. If left untreated, it has the potential to cause a complete loss of vision. With the rising incidence of diabetes, the significance of DR has also increased. As per global estimates, it is expected that more than 600 million people will be diabetic by the end of the year 2040 [29]. Epidemiological studies have shown that DR affects one in every three patients with diabetes and is the most common cause of blindness worldwide [30]. Therefore, early detection of DR and timely intervention are very important in order to prevent the ocular complications associated with this disease. AI technology is of great significance in effective screening for DR. By adopting AI-based applications, ophthalmologists can readily utilize this technology in conjunction with pre-existing clinical tools [31]. Currently, AI-based approaches have emerged as promising tools to automate the detection and grading of diabetic retinopathy.

Deep learning algorithms, specifically convolutional neural networks (CNNs), have remarkable performance in evaluating retinal images and identifying DR-related issues [32]. Several studies have shown that AI algorithms can achieve a level of sensitivity and specificity in detecting DR that is comparable to that of experienced professionals. DL can be used to detect DR by evaluating retinal fundus photographs, with a sensitivity of 97.5% [33]. Shah et al. developed an AI algorithm to detect DR by utilizing 112,489 images of the retina of diabetic patients. A very high sensitivity and specificity of 99.7% and 98.5%, respectively, were observed. The authors recommended the routine use of AI in the detection of DR [34]. AI-driven DR screening programs have a great potential to revolutionize the delivery of eye care services by providing prompt and reliable diagnosis, especially in resource-limited settings with limited access to expertise [35-37].

A system of DL, developed by Google AI Healthcare, derived disease characteristics from 128,175 retinal images that were graded by 54 experienced ophthalmologists. This AI system had the potential to swiftly identify various clinical signs of DR with high accuracy [38]. Ting et al. developed a DL system using clinical data from the Singapore Integrated Diabetic Retinopathy Program and external ophthalmological data sets from six different countries, including China, Hong Kong, Singapore, the USA, and Australia. The sensitivity and specificity of this DL system were 90.5% and 91.6%, respectively, with overall accuracy reaching 93.6% [7]. Tufail et al. analyzed three different automated DR image assessment systems (ARIAS), namely “EyeArt” (Eyenuk Inc., Woodland Hills, United States of America), “Retmarker” (Retmarker SA, Meteda Group, Taveiro, Portugal), and “iGradingM” (Medalytix Ltd., Manchester, United Kingdom). It was observed that the sensitivity of EyeArt in diagnosing proliferative retinopathy was 99.6% and that of Retmarker was 97.9% [39]. AI algorithms based on more than 30,000 retinal images acquired from the English Diabetic Eye Screening Programme (DESP) showed that by using AI tools, the workload of ophthalmologists can be reduced by more than 50% [40]. This is particularly useful in situations where reliable results are required in a shorter time duration.

Autonomous and assistive systems for diabetic retinopathy (DR) have matured from algorithm development to prospective deployment. Multiethnic validation demonstrated high diagnostic accuracy for referable DR detection from fundus images [1]. In a cluster randomized trial in urban safety-net clinics, embedding an autonomous DR system increased timely eye-care follow-up among youth with diabetes, showing that service-level outcomes improve when AI is wired into care pathways [41]. Prospective Thai clinic data further illustrate that real-time screening is workable, though careful attention to workflow and image acquisition is essential [3]. Systematic reviews confirm wide performance ranges across studies, reinforcing the need for external validation and transparent reporting [42].

Acquisition Details

Reported camera and protocol materially affect sensitivity and ungradable rates. The pivotal autonomous system used a single desktop non-mydriatic camera with a 45° field of view and specified capture criteria [2]. Multinational validation work aggregated images across several non-mydriatic cameras and care settings, improving generalizability but introducing device heterogeneity that must be stated [1]. Prospective deployments show that workflow, mydriasis, and real-time feedback all modulate quality and throughput [3,24]. Handheld imaging is feasible but remains quality-limited without rigorous capture guidance [25]. These devices and acquisition details are summarized in Tables 3, 4.

**Table 3:** Device and acquisition details DR: diabetic retinopathy, AUC: area under the curve.

Study	Device and field	Mydriasis	Setting	Notes relevant to performance	Limitations
Abràmoff et al. [2] pivotal autonomous DR	Topcon NW400, non-mydriatic, 45°	No	Primary care clinics	Pre-specified operating point; device-specific workflow and quality control	Device-specific training; no handheld images; external validation limited to predefined settings; calibration reported at a single operating point
Ting et al. [1] multiethnic validation	Multiple non-mydriatic cameras, typically 45°	Mixed	Screening programs and clinics	High AUC across populations; device heterogeneity requires disclosure	Device-heterogeneous pool; retrospective labels; no prospective outcomes; limited calibration reporting
Ruamviboonsuk et al. [3] prospective deployment	Desktop non-mydriatic, 45°	Mixed with reflex dilation	National screening programme	Real-time feedback reduced ungradable rates; operational lessons	Programme-specific workflow; mixed dilation policy; generalizability to handheld devices unknown
Mehra et al. [24] teleophthalmology	Desktop non-mydriatic	Reflex dilation protocol	Hospital clinics	Dilation policy lowered ungradable rates	Single-centre; operational co-interventions; outcome follow-up short
Salongcay et al. [25] handheld screening	Handheld fundus camera	Usually no	Community screening	Feasible; sensitivity depends on capture guidance and quality control	Sensitivity depends on capture guidance, a smaller field of view, and operator variability

**Table 4:** Representative performance of fundus-based systems for referable diabetic retinopathy DR: diabetic retinopathy, RCT: randomized controlled trial, PDR: proliferative diabetic retinopathy, DESP: diabetic eye screening programme.

Study	Setting	Device	Endpoint	AUC	Sensitivity	Specificity	External validation	Calibration reported
Abràmoff et al. [2] pivotal autonomous system	Primary care	Single desktop non-mydriatic, 45°	Referable DR	—	87–88%	89–91%	Prospective, USA	Operating point pre-specified
Ting et al. [1] multiethnic validation	Screening clinics	Multiple non-mydriatic, ~45°	Referable DR	0.93	90.5%	91.6%	External, Asia/US/AU	Not routine
Tufail et al. and Heydon et. al [39, 40] EyeArt in screening programmes	National DESP, UK	Mixed desktop cameras	Referable DR and PDR	—	up to 99.6% for PDR	—	Prospective	Not routine
Ruamviboonsuk et al. [3] Prospective national deployment	Multisite clinics	Desktop, real-time feedback	Referable DR	—	—	—	Prospective, Thailand	Not routine
Wolf et al. [41] Cluster RCT in youth	Safety-net clinics	Desktop non-mydriatic	Follow-up after abnormal screen	—	—	—	Prospective RCT	Not applicable

Limitations and Typical Failure Modes

Headline metrics attenuate with poor image quality, capture outside the trained field of view, device change between development and use, or noisy labels. Prospective and real-world deployments document failure modes tied to ungradable images, clinic workflow, and population shift [3,26]. External validation and transparent device reporting reduce these risks. Calibration and decision-curve analysis are infrequently reported, limiting clinical interpretability [1]. Table 5 gives a comparison of studies.

**Table 5:** Representative tele-ophthalmology and autonomous artificial intelligence studies RCT: randomized controlled trial, DR: diabetic retinopathy, AUC: area under the curve.

Study	Setting	Design	Ourcome	Take-home message	
Wolf et al. [41]	Primary care clinics	Cluster RCT of autonomous DR screening	Higher timely follow-up for abnormal results vs usual care	Workflow-aware deployment can change real-world behavior
Ruamviboonsuk et al. [3]	Hospital clinics in Thailand	Prospective, real-time DR screening	Feasible deployment with operational lessons	Capture workflow and operator training matter
Ting et al. [1]	Multicenter development and validation	Retrospective and prospective cohorts	High AUC for referable DR across devices and ethnicities	Emphasizes generalizability and bias checks
Zhelev et al. [42]	Systematic review	Meta-analytic synthesis	Wide accuracy ranges across systems	External validation and standard reporting needed

Uveitis

A comprehensive field overview concluded that uveitis is a suitable target for artificial intelligence across four domains: imaging-based anterior chamber cell quantification, phenotype and complications classification, treatment decision support, and prognostic modeling [43]. The strongest empirical progress to date is in quantitative anterior chamber cell counts on anterior segment optical coherence tomography, which correlate with Standardization of Uveitis Nomenclature grading and enable continuous, operator-independent measures [44-46].

Uveitis is well suited to objective, imaging-based quantification. Automated anterior segment - optical coherence tomography (AS-OCT) analysis can detect and count hyper-reflective particles in the anterior chamber and correlate with standardization of uveitis nomenclature (SUN) grading, offering a continuous, operator-independent measure of inflammation [44,45,47]. Pediatric feasibility is especially relevant because slit-lamp examinations can be difficult in children. A pilot study using swept-source AS-OCT reported strong correlations with SUN grading and proof-of-principle continuous grading [44]. More recently, algorithms have been validated against human counts and clinical activity in uveitic eyes, with work extending to transformer-based 3D approaches [45,46]. The clinical pipeline is also evolving toward imaging-based screening for juvenile idiopathic arthritis (JIA)-associated uveitis; the UVESCREEN1 randomized feasibility study protocol lays the groundwork for a pragmatic comparison of imaging versus routine clinical checks [48]. Table 6 summarizes how AI is being used for uveitis imaging and pediatric screening.

**Table 6:** AI for uveitis imaging and pediatric screening AC: anterior chamber, AS-OCT: anterior segment - optical coherence tomography, SUN: standardization of uveitis nomenclature, CV: cross-validation, AJO: American Journal of Ophthalmology, 3D: three-dimensional, JIA: juvenile idiopathic arthritis.

Reference	Task	Modality and cohort	Approach	Key results	External validation
Keino et al. [44]	AC cell quantification in mixed uveitis	AS-OCT, 31 patients, 51 eyes	Automated spot detection and continuous SUN scale	Spearman rho 0.774–0.843 vs SUN; leave-one-out rho 0.748	Internal CV
Pillar et al. [45]	AC cell quantification and validation	Swept-source AS-OCT in uveitis	Automated quantification validated against clinical grading	Reported agreement with clinical measures; details in AJO open-access	External dataset available in paper
Cifuentes-González et al. [46]	Advanced 3D cell quantification	3D AS-OCT in uveitis	Vision transformer for 3D cell quantification	Methodological advance; clinical correlation reported	To be expanded
Dave et al. [48]	Pediatric screening agenda	Children with JIA	Randomized feasibility protocol comparing imaging-based screening vs routine care	Trial design completed; feasibility endpoints specified	Multicenter

Limitations and Typical Failure Modes

Pediatric cooperation and variability in the standardization of uveitis nomenclature reference standard complicate training and evaluation. External validation remains limited, and device protocols differ across centers [44,46]. Table 7 summarizes gaps in the evidence.

**Table 7:** Where artificial intelligence is working now and where it is thin AC: anterior chamber, AS-OCT: anterior segment - optical coherence tomography, 3D: three-dimensional.

Representative sources	Domain	Current best evidence	Typical gaps to report	
Keino et al., Pillar et al., Cifuentes-González et al. [44-46]	AC cell quantification	AS-OCT automated counts correlate with Standardization of Uveitis Nomenclature grading; transformer approaches extend to 3D volumes	Limited multicenter external validation; device protocols vary; pediatric performance under-studied
Nakayama et al. [43]	Phenotype and complications	Review-level evidence supports feasibility for uveitic macular edema and complication screening	Heterogeneous endpoints; few prospective evaluations
Nakayama et al. [43]	Decision support	Early pipelines described	Lack of calibration and decision-curve analysis
Nakayama et al. [43]	Prognosis	Exploratory models in small cohorts	Need standardized outcomes and independent replication

Diagnosis and monitoring of glaucoma

Glaucoma is a progressive optic neuropathic condition that results in the cupping of the optic disc and the apoptosis of retinal ganglion cells. It is a leading cause of irreversible blindness and affects more than 3.5% of individuals between 40 and 80 years of age [49]. The incidence is rapidly increasing, especially in the context of increasing life-spans across the world, and it is estimated that by the year 2040, the number of individuals suffering from glaucoma is expected to exceed 112 million [50]. Early diagnosis and timely management of glaucoma are essential to prevent ocular complications. Differentiating between primary open-angle glaucoma (POAG) and chronic primary angle closure glaucoma (CPACG) can also be challenging. Manual assessment of the optic disc is time-consuming, subjective, and requires expertise in fundus examination. AI technology can be used to aid in the diagnosis of glaucoma using DL algorithms developed on images obtained through optical coherence tomography (OCT) [29]. Device and protocol matter for both fundus-based and anterior-segment pipelines; we specify a camera or AS-OCT/UBM platform and angle-assessment protocol where studies reported them [51-53].

Implementation of AI tools could provide a road map for cost-effective screening programs that could detect glaucoma from fundus images. Taj et al. reported an excellent performance of deep CNN architectures in identifying glaucomatous optic neuropathy through fundus images. The adoption of the Inception-v3 architecture resulted in high accuracy with an AUC of 0.98, a sensitivity of 99.1%, and a specificity of 99% [54]. The fundus images are based on a 2D imaging model that observes the surface of the optic nerve. An AI-based 3D image system can further enhance the diagnostic accuracy of these modalities. Lin et al. discussed the training of a 3D planning system by using 6,921 spectral domain OCT volumes. The results of these 3D systems showed an accuracy of 96% in detecting glaucomatous optic neuropathy (GON) and provided evidence of the excellence of the performance of these DL systems in interpreting fundus images [55].

Various 3D system models have great features to detect GON and have been shown to provide results comparable to those of trained glaucoma specialists [56]. Fu et al. developed a disc-aware ensemble network (DENet) based on more than 6,000 images from the Online Retinal Fundus Image Database for Glaucoma Analysis and Research (ORIGA), Singapore Chinese Eye Study (SCES), and Singapore Indian Eye Study (SINDI) datasets. An AUC of 0.91 showed the high accuracy of DENet in the automated detection of glaucoma through OCT images [57]. AI-based devices can also be employed to predict future visual loss in glaucoma patients. Hogarty et al. reported that AI technology could identify longitudinal glaucoma development and could be reliably applied to predict glaucoma progression with the help of ML [10]. Wang et al. suggested a DL approach to detect low visual field variability in glaucoma patients. The accuracy of the DL system based on data derived from a single visit to the clinic was 73%, thus pointing toward a possible role of DL in predicting visual field losses in glaucoma patients [58]. Besides this, the AI approach has a direct positive influence on the clinical assessment of different methods to identify glaucoma in suspected patients [59].

Limitations and Typical Failure Modes

Angle definitions differ across datasets, device geometry varies, and class imbalance is frequent. Image quality and ethnic or device domain shift reduce performance when models move between centers. External multi-center validation and explicit angle-assessment protocols are essential [26,51-53].

Anterior Chamber Angle Assessment and Primary Angle Closure Disease

Gonioscopy is the clinical reference for angle assessment, but it is operator-dependent and difficult to scale. Artificial intelligence models applied to anterior segment optical coherence tomography and ultrasound biomicroscopy learn anatomical features associated with primary angle-closure disease, including plateau iris configuration, and can classify angle status at the eye level. A recent multicenter study reported accurate recognition of primary angle-closure diseases on anterior segment optical coherence tomography using a deep learning pipeline, while earlier work showed prediction of plateau iris features and classification in suspects across devices. Ultrasound biomicroscopy supports lens and ciliary body visualization; deep learning on ultrasound biomicroscopy images has also achieved robust identification of primary angle-closure disease parameters. Device and protocol should be reported because performance varies with scan geometry, illumination, and population spectrum.

Limitations include heterogeneity in angle definitions, device geometry, and case mix; explicit reporting of device, protocol, grader agreement, and external validation is recommended for clinical translation [51,53,60]. Refer to Table 8 for a summary of studies on the use of AI for anterior chamber angle assessment.

**Table 8:** AI for anterior chamber angle assessment AS-OCT: anterior segment - optical coherence tomography.

Study	Modality and cohort	Task	Headline results	External validation	Notes and limitations
Yao et al. [60]	AS-OCT, multicenter clinical cohort	Recognize primary angle-closure diseases	Reported high diagnostic performance for spectrum of primary angle-closure diseases	Multicenter	Endpoint definitions and device details should be reported for transferability
Wanichwecharungruang et al. [51]	AS-OCT, clinic cohort	Predict presence of plateau iris	Accurate prediction of plateau iris configuration from AS-OCT	Single center	Angle definition protocol varies; prospective outcomes not assessed
Fu et al. [52]	AS-OCT, suspects	Analyze primary angle-closure suspects	Deep models discriminated suspects; study reported feature importance	Not clearly reported	Device mix unclear; threshold not pre-specified
Li et al. [53]	Ultrasound biomicroscopy, clinical cohort	Identify and quantify primary angle-closure parameters	Automated anterior segment identification and parameter assessment	External test set reported	Image quality variability and class imbalance noted

Visual electrophysiology

Machine learning applied to electroretinography (ERG) can classify disease states and estimate functional status from waveform features. In early glaucoma, ERG time-frequency features combined with supervised learning achieved promising discrimination in research settings [61]. Synthetic data generation for ERG has also been explored to balance classes and improve model performance, although this remains developmental [62]. While clinical deployment is not ready, the direction of travel is clear for functional biomarkers. Table 9 summarizes these findings.

**Table 9:** AI for visual electrophysiology ERG: electroretinography, ML: machine learning.

Reference	Task	Dataset	Method	Key finding
Gajendran et al. [61]	Early glaucoma classification from ERG	Clinical ERG signals	ML on wavelet and time-frequency features	Discriminative performance in early disease; research-grade study
Kulyabin et al. [62]	Augment ERG data to improve classifiers	Human light-adapted ERGs	Synthetic waveform generation plus ML classifiers	Improved minority-class performance in balanced accuracy

Age-related macular degeneration

Age-related macular degeneration (AMD) is a major cause of vision impairment in older adults. It refers to senile degenerative changes in the macula, which are responsible for almost 9% of all cases of blindness [63]. It is estimated that approximately 288 million individuals worldwide are facing the problem of AMD, and the incidence is expected to rise by another 10% by the end of the year 2040 [63]. The Age-Related Eye Diseases Study (AREDS) has classified AMD into different categories based on severity, and the American Academy of Ophthalmology suggested that individuals with intermediate AMD must have ophthalmological consultations once every two years [64]. This presents greater concerns about the elderly population, as they require frequent referrals to ophthalmological clinics. Where reported, we list OCT vendor, scan protocol, and referral thresholds because performance shifts between devices and datasets have been observed in prospective and translational studies [4].

Similar to other ocular conditions, DL systems can serve as a valuable tool in screening these patients for further evaluation. According to Ting et al., clinically acceptable DL systems, particularly those trained on the clinical findings and images from commonly used tests to detect AMD, can identify referable cases of AMD [7]. In multiethnic research on data from the Singapore National Diabetic Retinopathy Screening Program, more than 72,000 fovea-centered images of AMD patients were incorporated into the DL system. The accuracy of the DL system was compared with that of expert human graders. The AUC was 0.93, with a sensitivity and specificity of 93.2% and 88.7%, respectively [1]. The success of DL systems in detecting AMD implies that these systems may be used routinely for screening in the elderly population. Table 10 summarizes these findings.

**Table 10:** Selected OCT-based systems for referral triage or AMD classification OCT: optical coherence tomography, AUC: area under the curve, AMD: age-related macular degeneration.

Study	Modality	Task	AUC	External validation	Calibration
De Fauw et al. [4]	OCT + referral pathways	Urgent referral triage	≥0.98 for urgent pathway	External (Moorfields test sets)	Reported
Ting et al. [1]	Fundus	Referable AMD	0.93	External, multiethnic	Not routine

Intraocular tumors and ocular oncology

Deep learning applied to color fundus photographs can help differentiate benign choroidal nevi from small melanomas and may complement clinician risk scores such as mushroom shape, orange pigment, large size, enlargement, and subretinal fluid (MOLES), and to find small ocular melanoma using helpful hints daily (TFSOM-UHHD). A recent multicenter study of 802 images achieved AUCs of 0.88 in two independent test cohorts and showed higher sensitivity than resident and consultant ophthalmologists, while performing comparably to ocular oncologists [65]. Self-supervised frameworks and machine-learning models predicting nevus transformation are emerging, although they require prospective evaluation and standardized pathways for escalation to ocular oncology teams [66,67]. Integration with multimodal imaging and formal risk scores should be explicit to avoid over-reliance on single-modality outputs. Table 11 summarizes these studies.

**Table 11:** Artificial Intelligence for ocular oncology triage CNN: convolutional neural networks, AUC: area under the curve, MOLES: mushroom shape, orange pigment, large size, enlargement and subretinal fluid, TFSOM-UHHD: to find small ocular melanoma using helpful hints daily, ML: machine learning

Reference	Clinical question	Modality and dataset	Model	Performance	Comparator
Sabazade et al. [65]	Differentiate small melanoma vs nevus	Fundus photos, 802 images, multicenter	CNN with segmentation-assisted pipeline	AUC 0.88 in two test sets; 100% sensitivity and 74–81% specificity at chosen threshold	Outperformed MOLES and TFSOM-UHHD by AUC; comparable to ocular oncologists
Jackson et al. [67]	Differentiate nevus vs melanoma	Fundus photos, multi-site	Self-supervised representation learning	High discriminatory performance reported; needs prospective validation	Multiple clinician comparators
Tailor et al. [66]	Predict transformation risk	Retrospective clinical cohort	Classical ML on clinical features	Predictive utility for transformation proposed	—

Limitations and Typical Failure Modes

Case mix, center bias, and prevalence shift influence apparent accuracy. Referral thresholds and oncologist comparators must be stated. Prospective follow-up for transformation risk is still uncommon [65-67].

Cataract and anterior segment disorders

According to the World Health Organization (WHO), cataract is the leading eye disease, affecting more than 65 million people worldwide. For this reason, cataract surgery is the most commonly performed ophthalmological operation [68]. The use of AI has provided great services and emerged as a valuable tool that could help eye surgeons plan cataract surgery. One of the key challenges in cataract surgery planning is the prediction of effective lens position in the eye that could be determined with the help of the refractive power of the implanted intraocular lens (IOL). ML algorithms, based on large data sets containing information about corneal topography and post-operative results, help estimate the ideal power of lenses and different surgical parameters to reduce the need for post-operative arrangements [69, 70]. Evaluation of corneal topographic data helps to identify the aberrations and irregularities that could impact the outcomes related to cataract surgery.

The use of ML models spans various phases of cataract management. They can be utilized for pre-op screening, IOL power calculation, e.g., application of the Kane formula through AI-based approaches, keratometry, and calculation of axial length and anterior chamber depth. Intra-operative AI devices such as “Touch Surgery Enterprise” can assist in data analysis and play a role in clinical teaching [71]. ML models help assist surgeons and demonstrate greater predictive accuracy as compared to other traditional methods of IOL power calculation, particularly in the complex biometry of refractive surgery. Despite such advancements, the challenges are also increasing with the adoption of AI-driven technology. Direct surgery planning systems require proper validation through studies examining different patients, populations, and surgical settings that could provide comprehensive information about the integration of the AI algorithms into the existing framework of ophthalmological services [72].

There is emerging evidence favoring the use of AI in anterior segment conditions as well. These conditions include infective keratitis, keratoconus, angle closure glaucoma, iris tumors, corneal transplants, and patients undergoing refractive surgery [73]. The use of multiclass computer-aided systems designed through ML using various algorithms can help in the diagnosis of various anterior segment abnormalities. Cai et al. developed a large-scale data set from more than 2,000 images of pathological conditions of the anterior segment of the eye. The image set was annotated through an AI-based annotation system named “EyeHealer”. The images were annotated at the pixel level, and the result showed that segmentation models based on these images outperformed models based on medical segmentation [74].

Intraoperative video analysis supports surgical phase recognition, instrument tracking, and skill assessment. For cataract surgery, public datasets now enable benchmarking and external validation. Cataract-1K is a large curated video set for scene segmentation, phase recognition, and irregularity detection, designed explicitly for clinical relevance and cross-domain tasks [75]. The older Cataract-101 set remains widely used for phase classification and active-learning approaches, and has supported modern temporal models [76-78]. These findings are summarized in Table 12. Prospective work should evaluate human-AI teaming in theaters and measure operative and patient outcomes.

**Table 12:** Surgical video artificial intelligence resources and performance

Reference	Use case	Dataset	Scope	Illustrative performance	Notes
Ghamsarian et al. [75]	Phase recognition, segmentation, irregularity detection	Cataract-1K	1,000+ cataract videos with multi-task labels	Benchmarks reported for phase recognition and segmentation	Designed for clinical tasks and domain adaptation
Müller et al., Schoeffmann et al. [76, 77]	Phase recognition	Cataract-101	101 phaco surgeries, 10 phases	Accuracy around 90% with modern temporal models on validation sets	Broad adoption for model development and active learning
Mahmoud et al. [78]	Automated intraoperative phase ID	CatStep study	Cataract surgery videos	High performance phase identification reported	Highlights translational potential

Cornea and external disease

Various corneal conditions can be effectively analyzed using AI models. Keratoconus detection strategies involve computer-aided diagnosis systems with algorithms demonstrating high accuracies ranging from 96% to 99% [79]. DL models have achieved sensitivities and specificities exceeding 90% and diagnostic accuracies up to 99% using AI tools such as “CorneaNet” [80,81]. Additionally, ML algorithms utilizing topographies such as “KeratoDetect” have achieved 99.3% accuracy in identifying keratoconus-related ectasia [82]. Similarly, topography-based algorithms have been used in predicting the safety and efficacy of surgical procedures such as small incision lenticule extraction (SMILE), laser in-situ keratomileusis (LASIK), and photorefractive keratectomy (PRK) with high accuracy [83-85]. Corneal dystrophies such as Fuchs dystrophy were detected using anterior segment OCT and specular microscopy with AUC 0.998 and 0.967, respectively [86,87]. Deshmukh et al. used slit-lamp techniques and images to detect granular stromal dystrophies with an accuracy of 95% using DL algorithms [88].

The condition of the corneal epithelium can also be reliably assessed using DL methods such as “U-net”. Heinzelmann et al. showed that the U-net can measure corneal epithelial cell counts with an accuracy identical to that of gold standard methods. Epithelial cell counts form corneal epithelia from 100 grafts were correlated between U-net and an experienced observer, and a statistically significant correlation coefficient of 0.9 (p < 0.0001) was observed [89]. Treder et al. utilized a DL-based classifier for the detection of Descemet's membrane endothelial keratoplasty (DMEK) graft dislocation and demonstrated a sensitivity of 98%, specificity of 94%, and accuracy of 96% using anterior segment OCT [90]. Another model, “VGG19”, evaluated the need for re-bubbling of detached grafts after DMEK, with an AUC of 0.96, a sensitivity of 96.7%, and a specificity of 91.5% [91]. Thus, the use of AI has the potential to transform corneal grafting and can be employed by corneal banks to reliably assess the condition of corneal grafts.

Endothelial integrity is pivotal for graft selection and post-keratoplasty outcomes. As shown in Table 13, AI models now quantify endothelial cell density (ECD), hexagonality, and polymegathism from specular microscopy, including challenging real-world images. A self-supervised segmentation model trained on more than 15,000 donor endothelial images achieved automated ECD estimation suitable for eye-bank workflows [92]. Additional deep models segment endothelial cells and distinguish Fuchs endothelial corneal dystrophy across staging, using specular microscopy and high-definition OCT [86,87,93]. Emerging widefield specular microscopy demonstrates promising pilot performance for detecting Fuchs dystrophy and may overcome the field-of-view constraints of conventional specular imaging [94].

**Table 13:** Artificial intelligence for corneal endothelium assessment and graft-relevant metrics ECD: endothelial cell density, FECD: Fuchs endothelial corneal dystrophy, OCT: optical coherence tomography, DL: deep learning, AUC: area under the curve.

Reference	Clinical task	Modality and dataset	Model/approach	Headline result	Notes
Benetz et al. [92]	Automated ECD quantification for eye-bank images	Specular microscopy; 15,138 images from 8,169 donors (two eye banks)	Self-supervised deep learning segmentation	Automated ECD estimation from real-world images; study reports scalability for quality control	Eye-bank deployment relevance
Shilpashree et al. [87]	Endothelium segmentation and morphology in FECD	Specular microscopy; FECD vs healthy	DL segmentation with morphology features	Differentiates FECD and healthy; enables perimeter- and cell-feature analyses	Supports pre- and post-keratoplasty assessment
Eleiwa et al. [86]	Early FECD detection and staging	High-definition OCT of cornea	DL classifier for early vs late FECD and healthy	High diagnostic performance for early FECD staging; image- and eye-level AUCs reported	OCT pathway complementary to specular
Foo et al. [94]	FECD detection from widefield endothelium imaging	Widefield specular microscopy	DL classifier	Pilot study showing reliable FECD detection; proof-of-concept for larger field	Addresses limited field of standard specular

AI algorithms have been employed for the diagnosis of dry eye disease as well. Algorithms such as multilayer perception (MLP) and support vector machines (SVM) have been applied for the interpretation of interference patterns in the lipid layer of tear film. The estimation of the length and width of meibomian glands through meibography images to differentiate diseased from healthy glands is effectively aided by a combination of scale-invariant feature transform (SIFT) and SVM algorithms [95]. Other algorithms used for the detection of dry eye disease include the Markov random field (MRF) and random sample consensus (RANSAC) algorithms [96]. Further utility of AI in external diseases was demonstrated by Habibalahi et al. in detecting ocular surface squamous neoplasia (OSSN). They compared histopathological biopsy specimens of OSSN with a custom-built, autofluorescent multispectral imaging system. The system could demonstrate a strong difference between neoplastic and normal tissue (p<0.0005), delineating a well-circumscribed neoplastic and non-neoplastic interface [97].

Limitations and Typical Failure Modes

Specular microscopy has field-of-view constraints and cell-border ambiguity. Many studies lack external validation on eye-bank or post-keratoplasty images. Reporting segmentation quality control and calibration would strengthen clinical translation [44,46,86,92].

Pediatric eye diseases

Pediatric ophthalmology, a challenging sub-specialty in ophthalmology, has increasingly been utilizing AI support due to the specific nature of this field, which includes younger patients and their associated ophthalmological problems [98]. Diagnosis in the pediatric population is often difficult due to a lack of cooperation by younger children and challenges with establishing verbal contact. Examination under general anesthesia is not always a favorable option. These challenges contribute to the diagnostic difficulty in pediatric patients, and the introduction of AI has thus served as a valuable addition to the field. Screening of children for visual acuity and identification of the cause of eye disorders form the mainstay of the practice in pediatric ophthalmology. Even a minor subjective variation in the assessment of the pediatric population can have a profound effect on treatment schedules [99].

Tackling the issue of subjective discrepancies in the diagnosis of conditions with complex clinical presentations can be done through the incorporation of AI tools. The “TrackAI” project, developed by Pueyo et al. to identify visual disturbances in children, includes two main components. Firstly, a novel test of vision is incorporated into a digital device known as Device for an Integral Visual Examination (DIVE), and secondly, various AI algorithms are employed to run on a smartphone to automatically analyze and interpret the visual data generated through DIVE [100]. Various other CNNs are already being used to screen and diagnose retinopathy of prematurity (ROP) among infants. Among these networks, “SenseNet-121” has shown promising results for screening, and “ResNet-18” has proven beneficial in the treatment planning of ROP [101]. Liu et al. combined both of these networks and observed a sensitivity of 70.6% and specificity of 94.1% in the identification and management planning of ROP in infants. Other pediatric conditions, such as pediatric cataract, can also be identified and staged through AI platforms such as “CC-Cruiser”. Although these AI platforms are in the initial stages of introduction and have limited accuracy, yet they have been shown to reduce the time to reach diagnosis and eliminate examination stress in younger patients [102].

Oculoplastics

The field of oculoplastic and reconstructive surgery of the eye typically includes cosmetic surgeries involving the orbit, eyelids, and lacrimal structures. This field heavily relies on analysis of ophthalmological images and, therefore, can particularly benefit from AI tools designed to analyze, interpret, and compare digital images [103]. These AI tools can extract parameters from images and help in screening, diagnosis, and management planning of various oculoplastic conditions. Van Brummen et al. used “PeriorbitalAI”, an automation tool to determine various static and dynamic parameters of eyelids, such as canthal height, intercanthal distance, and margin to reflex distance. The 95% confidence intervals of the measurements taken through AI automation tools showed a considerable overlap with those taken by human graders, thus exhibiting a comparable performance to human experts [104].

AI has been effectively employed in the management of ptosis, a very common presentation to the oculoplastic clinics. Chen et al. observed a strong correlation (r=0.91) between margin to reflex distance (MRD) assessed through AI and through current gold standards. It was concluded that AI tools can provide accurate reading in a convenient and time-saving manner [105]. Similarly, Thomas et al. used “OpenFace”, an AI tool designed to analyze facial landmarks in real time in patients with ptosis, and found an agreement of almost 95% between parameters estimated through OpenFace and those calculated by clinical experts [106]. Seth et al. explored the role of a chatbot (ChatGPT) in advanced oculoplastic research through asking various questions based on the technicalities of the field. The responses, although sufficient to answer queries of the general public, lacked depth and precision to serve as an alternative to clinical experts [107]. The use of the “ResNet” AI model in the detection of thyroid orbitopathy is 87.3% sensitive and 84% specific [108]. Additionally, the reconstructive surgery of eyelid and other ocular structures has been revolutionized by the use of 3D reconstruction images and the development of patient-specific implants (PSIs) [109]. Thus, the literature is evident on the beneficial role of AI in the field of oculoplastics. 

Neuro-ophthalmic conditions

Deep CNNs applied to fundus images have allowed the detection of optic disc pathologies, differentiating them from normal optic disc findings. The Brain and Optic Nerve Study with Artificial Intelligence (BONSAI), conducted in Singapore, analyzed 6443 fundus images and accurately distinguished papilledema from normal optic disc with an AUC of 0.98, papilledema from ischemic optic neuropathies with an AUC of 0.95, and true papilledema from false papilledema with an AUC of 0.94 [110]. Interestingly, a head-to-head comparison reported the time needed for the BONSAI-DL system to be 25 sec vs 61 min and 74 min for 2 expert neuro-ophthalmologists in grading 800 new fundus images for papilledema and other optic disc abnormalities [111]. They also compared the DL system with the FOTO-ED study, which previously demonstrated a poor recognition of fundus findings by ED physicians, and demonstrated an AUC of 0.92, sensitivity and specificity of 75.6%, and 89.6%, respectively [112]. The same DL system, when applied to the pediatric population, has demonstrated an accuracy close to 90% in the grading of papilledema in comparison to expert neuro-ophthalmologists [113,114]. Other studies using different learning systems and datasets also demonstrated accuracy with a range of 85-99% in detecting the presence of papilledema, and grading of papilledema with an accuracy of 97-99% [115-117].

Ophthalmological emergencies

Traumatic and non-traumatic ocular emergencies are fairly common in clinical practice. Only in the United States, around 2-3 million cases of ocular emergencies present in the accident and emergency departments each year [118,119]. Various mobile applications and AI tools have been developed to assist patients in categorizing their disease severity and advising if they require an emergency visit to a healthcare facility. The accuracy of these applications in reaching a diagnosis ranges from 39% in emergency cases to 88% in non-emergency cases [120]. However, a South Korean study on 1,681 patients with ocular emergencies showed that AI models based on algorithms designed on minority oversampling techniques can achieve an accuracy of 99.05% in categorizing emergency ophthalmological conditions based on severity [121].

The triage of patients presenting with ocular emergencies in the accident and emergency departments is the foremost step in the management of these patients. However, the presenting symptoms can often be misleading, and prioritizing these patients according to urgency to receive medical care can be subject to human error [122]. ML can be employed to safely triage these patients. Chen et al. developed an AI system called “EE-Explorer” to segregate the patients presenting with ocular emergencies according to the severity of the condition. It consisted of two distinct models: a “triage model” and a “primary diagnostic model”. These models were designed on a metadata of more than 4,000 ophthalmic images. The triage model outperformed the nurses working in the accident and emergency departments, with an overall diagnostic accuracy of 98.2% [123]. Similarly, data from 11,315 patients presenting to the emergency department of Moorfields Eye Hospital, London, UK, were utilized to build the DemDx Ophthalmology Triage System (DOTS). This ML system had a similar sensitivity and a 17.3% higher specificity than trained nurses in prioritizing patients presenting with ocular emergencies [124]. The success of these ML systems points toward a definitive role for AI in the management of ophthalmological emergencies.

Image quality, device heterogeneity, and acquisition dependencies

Image quality strongly conditions the performance and safety of computer vision models in retinal screening and beyond. Prospective deployments show that capture workflows, mydriasis, handheld imaging, and real-time feedback all modulate ungradable rates and downstream outcomes [3,25,41]. Reviews of public retinal datasets also reveal inconsistent or weak image-quality criteria, which undermine generalizability and external validity [125,126]. The practical remedy is twofold: incorporate robust image-quality assessment at the point of capture and specify minimal acquisition standards in clinical protocols. Tables 14, 15 summarize these findings.

**Table 14:** Practical Considerations for Image quality in Ophthalmic AI DR: diabetic retinopathy, AI: artificial intelligence.

Reference	Scenario	Key finding	Practical mitigation
Ruamviboonsuk et al. [3]	Real-time DR screening in clinics	Prospective deployment feasible with point-of-care adjudication; workflow matters	Real-time feedback to operators, clear referral thresholds
Salongcay et al. [25]	Handheld fundus imaging	AI can grade referable disease from handheld images; image quality remains a limiter	Train with handheld data, enforce capture criteria, repeat low-quality images
Mehra et al. [24]	Mydriasis vs non-mydriasis	Mydriasis reduces ungradable rates in teleo-phthalmology programs	Use mydriasis for screening when feasible or implement capture retry logic
Gonçalves et al., Liu et al. [125,126]	Public dataset quality	Many datasets lack rigorous, standardized quality labels	Add automated quality control and publish quality protocols

**Table 15:** Modality-specific limitations and what to report DR: diabetic retinopathy, QC: quality control, OCT: optical coherence tomography, AMD: age-related macular degeneration, AS-OCT: anterior segment - optical coherence tomography, UBM: ultrasound biomicroscopy, SUN: standardization of uveitis nomenclature.

Representative sources	Modality	Common sources of bias/error	Minimum reporting to add
Ting et al., Abràmoff et al., Ruamviboonsuk et al., Mehra et al., Salongcay et al., Beede et al., Zhelev et al. [1-3,24-26,42]	Fundus DR screening	Ungradable images, device/domain shift, spectrum bias	Camera model, field of view, mydriasis policy, QC threshold, external test set, calibration
De Fauw et al., Beede et al. [4,26]	OCT for AMD triage	Scanner vendor/protocol, referral threshold drift, label noise	Vendor, scan protocol, referral rule, calibration, clinical outcome where available
Beede et al., Wanichwecharungruang et al., Fu et al., Li et al. [26,51-53]	Glaucoma from fundus/AS-OCT/UBM	Variable angle definitions, device geometry, class imbalance	Device and angle protocol, grader agreement, external validation
Keino et al., Cifuentes-González et al., Eleiwa et al., Benetz et al. [44,46,86,92]	Corneal endothelium (specular/OCT)	Field-of-view constraints, cell border ambiguity	Imaging modality, preprocessing, segmentation QC, external test set
Keino et al., Cifuentes-González et al. [44,46]	Uveitis AS-OCT	Pediatric cooperation, reference standard variability	SUN reference, device, grader protocol, external validation
Sabazade et al., Tailor et al., Jackson et al. [65-67]	Ocular oncology (nevus vs melanoma)	Case mix, center bias, prevalence shift	Imaging protocol, referral threshold, oncologist comparator, prospective follow-up

Challenges and limitations of AI in ophthalmology

Across subspecialties, headline performance commonly decreases with domain shift, device change, and real-world image quality issues. Few studies report calibration or decision-curve analysis, and prospective external validation remains the exception rather than the rule. Given heterogeneity in devices, thresholds, and clinical endpoints, a hierarchical meta-analysis of diagnostic accuracy would require a narrow, pre-specified subset. We therefore opted for a comparative narrative synthesis and reported effect estimates with 95% CIs where provided, consistent with PRISMA 2020 and standard approaches to test-accuracy synthesis.

The uses and applications of AI technology in the field of ophthalmology have been discussed. Although the introduction of AI has revolutionized the practice of ophthalmology with innumerable benefits, there are a number of challenges that ophthalmologists face when utilizing this technology. The primary issue with the wide-scale implementation of AI technology in any medical field is ethical considerations. There is still a lack of trust among patients, the main stakeholders, in the general use of AI technology for clinical management. For this reason, the health industry lags behind other industries in the utilization of AI tools [127]. Additionally, there are concerns about breaches of data privacy, which have limited the use of AI in clinical settings. The development of DL systems requires large data sets of patients’ clinical characteristics. The data input and data output associated with these DL systems raise concerns about data privacy [128].

Another limitation to the acceptability of AI technology by patients is the impact on the doctor-patient relationship. The use of AI in ophthalmologic examinations tends to reduce the interaction time between ophthalmologists and patients and thus gives rise to concerns about patient safety [128]. It is also important to thoroughly validate the DL systems before employing them in ophthalmological practice. A large-scale, multicenter, non-interventional validation study on seven different AI algorithms for the diagnosis of DR showed that only two could perform better than human examiners, thus raising serious concerns about the accuracy of such algorithms [129]. It is, therefore, imperative to test these algorithms in real-world clinical settings before wide-scale implementation into ophthalmological practice. The disease trends exhibit periodic shifts, and a mismatch between training data and the operational requirements can occur, thus requiring recalibration from time to time [130]. It is therefore essential to keep the algorithms clinically relevant and updated by incorporating new data and trends. Finally, there are also issues with the training of ophthalmologists in using AI tools, patients’ engagement, a lack of regulatory mechanisms, and the financial and legal implications of the development and implementation of AI technology that hinder the use of AI technology in the field of ophthalmology [131]. 

Future of AI in ophthalmology

Currently, AI technology is being used for screening, diagnosis, and treatment planning for various ocular pathologies, such as DR, cataracts, AMD, and glaucoma. Some of the common ready-to-use AI tools for ophthalmological practice include “Optovue iWellness”, “IDx-DR”, and “Eyenuk EyeArt” for diagnosis and screening of diseases involving the retina, particularly DR, “CIRRUS HD-OCT with AngioPlex” for assessment of retinal blood flow, and “Topcon Harmony with OphtAI” for fundal imaging [132-134]. Certain other open AI tools that are readily available online and free of cost include “RetinaNet”, “DeepDR”, “Ilastik”, “VGG-16”, “OpenDR”, “TensorFlow”, “ResNet50”, and “PyTorch” [134-136]. However, most of the DL algorithms being used in these AI tools are still in their infancy stages, and further research in real clinical settings is needed for their validation.

Clinical translation efforts by ophthalmologists and AI researchers are needed in order to develop cutting-edge AI technology to solve clinical problems in the field of ophthalmology [137]. The sensitivity and specificity of these DL systems should be enhanced by improving the quality of the data, sample size, heterogeneity, and ultimately the generalizability of the training data sets [138]. There are also ethical and moral concerns related to AI technology that have been discussed elsewhere in this review. The wide-scale implementation of AI technology is only possible once these ethical issues have been adequately addressed and regulatory mechanisms are in place for effective implementation of AI technology in the field of ophthalmology [139]. Finally, there is a need to develop guidelines for the effective use and operationalization of AI technology and to engage all the relevant stakeholders to enhance their acceptance of the adoption of newer AI technologies in daily clinical practice. The future of AI technology in the field of ophthalmology appears bright, and with continuous efforts, it can have far-reaching benefits for patients suffering from ocular diseases.

Education is a near-term win. For clinicians, annotated surgical video datasets now support deliberate practice, phase timing, and objective feedback outside the operating room [75-78]. For patients, deployment trials of autonomous DR screening demonstrate how immediate, comprehensible outputs can catalyze follow-up behavior in underserved settings [41]. Future studies should evaluate co-designed patient-facing explanations that are accurate, culturally appropriate, and linked to action.

## Conclusions

The integration of AI technology in different aspects of ophthalmology has demonstrated great potential for improved patient care. Various studies have shown that AI can provide early detection of diabetic retinopathy, age-related macular degeneration, cataracts, and pediatric conditions. It can also help in the triage of emergency conditions and identify the need for referral to ophthalmology clinics. There is substantial evidence to advocate for the role of AI in personalized surgery planning, particularly in oculoplastics and reconstructive surgery. AI technologies have the potential to provide innovative solutions to deal with long-term challenges. Our literature review highlighted the remarkable developments made in the integration of AI algorithms into clinical practice for the implementation of various tasks such as risk detection, stratification, and treatment plans. Many AI-driven systems have demonstrated greater accuracy and efficiency in analyzing data to diagnose or predict ocular conditions as compared to human experts. This can result in swift decision-making, better outcomes, and improved healthcare delivery.

However, large-scale adoption of AI technology in ophthalmology is not without limitations. There are challenges related to data quality, validation, ethical issues, data privacy, and regulatory frameworks that must be carefully addressed to make sure that medical professionals are effectively implementing AI technology in clinical practice. Moreover, there is a need to develop collaboration and consistent research that could define the optimized use of AI algorithms and integrate this technology into existing ophthalmological services. Although many studies have been conducted on this topic, there is still much work to be done, and the authors recommend future research in this domain, particularly in the context of developing countries. It is essential to ensure ongoing innovation and regulatory support to transform the landscape of patient care, which will result in improved outcomes and quality of life for the patients who are affected by eye diseases.
